# Rational Fabrication of Ag Nanocone Arrays Embedded with Ag NPs and Their Sensing Applications

**DOI:** 10.1021/acsomega.2c05854

**Published:** 2022-12-06

**Authors:** Hongxu Chen, Xing Li, Yu Wang, Yan Li, Yingfeng Yu, Haidong Li, Baoqing Shentu

**Affiliations:** † College of Material and Textile Engineering, Jiaxing University, Jiaxing 314001, China; ‡ State Key Lab of Chemical Engineering, Department of Chemical and Biological Engineering, Zhejiang University, Hangzhou 310027, China; § Zhejiang Yuhua Timber Co., Ltd., Jiaxing 314101, China

## Abstract

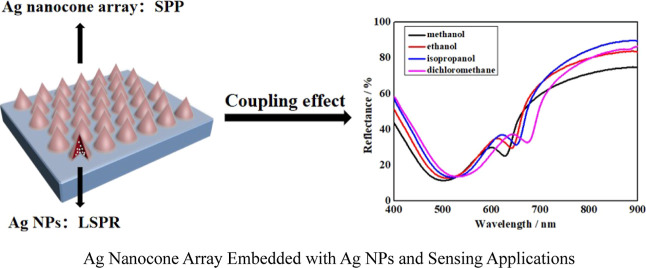

Colloidal lithography is used to design and construct a high-performance plasmonic sensor based on Ag nanocone arrays embedded with Ag NPs. The surface plasmon polariton (SPP) of the Ag nanocone array and the localized surface plasmon resonance (LSPR) of Ag NPs inside the nanocones can both couple incident photons. Sharp reflectance troughs are considerably enhanced by coupling the SPPs and LSPR, which is made possible by carefully tuning the nanocone sizes. To maximize the line shape and sensitivity, other geometric factors, such as the thickness of the silver layer and the size of the Ag NPs, are modified. Finite-difference time-domain computations confirm these hypotheses and experimental findings. We use well-researched solvents with various refractive indices as a model system to demonstrate good sensing performance as a proof of concept. The crystal used in this investigation has the ideal refractive index sensitivity, having 500 nm lattice constant, 350 nm nanocone height, and 350 nm base diameter (aspect ratio = 1). The Ag nanocone array embedded with Ag NPs is a good contender for a sensing platform due to its compact structure and efficient read-out apparatus.

## 1 Introduction

In recent years, with the development of nano-optics and photonics, the research on surface plasmons (SPs) of micro-/nanofunctional structures has attracted extensive attention, which have been widely used in the fields of enhanced transmission, fluorescence enhancement, as negative refractive index materials, and as optical waveguides.^1−9^ It is found that when the incident light wave irradiates on the surface of the metal micro-/nanoarray structure, the SPs with strong excitation can be excited at the metal/medium interface to significantly change the reflection characteristics of the metal surface, enhance the local near-field light wave of the metal surface, and modulate the surface resonance wavefield. The collective oscillations of the conduction electrons in metallic nanostructures are known as SP resonance (SPR).^10−12^ The size, shape, and structure of the nanostructures and the dielectric characteristics of the external environment significantly impact the SPR’s position and intensity.^13−18^ Plasmonic metallic nanostructures can be used to make optical sensors because of their wide range of response factors. Thus, the use of plasmon-enhanced optical sensors for analyte detection in biological diagnosis, homeland security, food safety, and environmental control is expanding.^19−30^

Research in chemistry, biochemistry, and biomedicine heavily relies on SP-based sensing technologies. SP polaritons (SPPs) and localized SPR (LSPR) are two different types of SPs.^31−35^ SPPs are charge oscillations that propagate on the surface of thin metal sheets. SPPs cannot be stimulated by free-space radiation and must rather be excited by momentum matching, such as periodicity in a nanostructure. The refractive index of the surrounding medium modulates SPPs, translating the signal from the sensor. In contrast to SPPs, LSPR happens when a metallic nanostructure’s dimensions are less than the wavelength of incoming light, causing surface electrons to oscillate collectively but not propagatively. The LSPR provides the foundation for colorimetric plasmonic sensors since it is highly dependent on the refractive index of the surrounding medium.

Nanostructured materials and architecture allow the customization of LSPR and SPP, which open up a wide range of design options for plasmon-enhanced sensors. Recently, some efforts have been made for plasmon-enhanced optical sensors. Many metal micro-/nanostructures, such as triangular nanoprism arrays, nanohole arrays, nanocrescent arrays, gold nanorods, and others, have been synthesized and studied.^36−45^ However, there have been few reports on integrating metal nanoparticles and metal micro-/nanoarray structures, which will benefit from studying the effects of both LSPR of metal nanoparticles and SPP of metal micro-/nanoarray structures simultaneously. Future plasmon-enhanced sensors will be successful if plasmonic materials and architectures are well designed and novel fabrication techniques are discovered. Hence, introducing new processes for making plasmonic materials and architectures using metal nanoparticles and metal micro-/nanoarray structures is crucial.

In this work, we developed and fabricated a sensor based on an Ag nanocone array embedded with silver nanoparticles (Ag NPs), which can achieve the combination of metal nanoparticles with metal micro-/nanoarray structures. Because of its innately powerful and acutely resonant properties, silver is the preferred fabricating material in usual circumstances.^46^ The significant potential to improve the electric field is discovered to be produced synchronously at Ag NPs and an Ag nanocone array. Optimizing the nanocone height and base diameter achieves a crisp and intense line shape. The coupling effect between the LSPR of the Ag NPs and the SPP of the Ag nanocone array, which is an important component of reflectance spectra, is realized by concentrating on the effect of the nanocone aspect ratio. Theoretical simulations employing finite-difference time-domain (FDTD) methods are used to confirm all experimental findings. Moreover, solvent detection is finally carried out on samples with various nanocone aspect ratios to investigate its capability as a superior sensor.

## 2 Experimental Section

### 2.1 Reagents and Chemicals

We acquired 500 nm monodispersed polystyrene (PS) nanospheres from Janus New Materials Co., Ltd. PCL. Silicon (100) wafers were washed using a concentrated 98% H_2_SO_4_/30% H_2_O_2_ (v/v: 7:3) solution at 80 °C for 4 h. After cleaning, the silicon wafers were continuously rinsed with Milli-*Q* water (18.2 MΩ cm) and ethanol before being dried using an N_2_ stream. From Beijing Chemical Works, we purchased sodium dodecyl sulfate, sulfuric acid, hydrogen peroxide, dichloromethane, methanol, isopropanol, and ethanol. Sigma-Aldrich provided the *p*-amino thiophenol (PATP).

### 2.2 Fabrication of the PAA Nanocone Array

**Figure 1:**
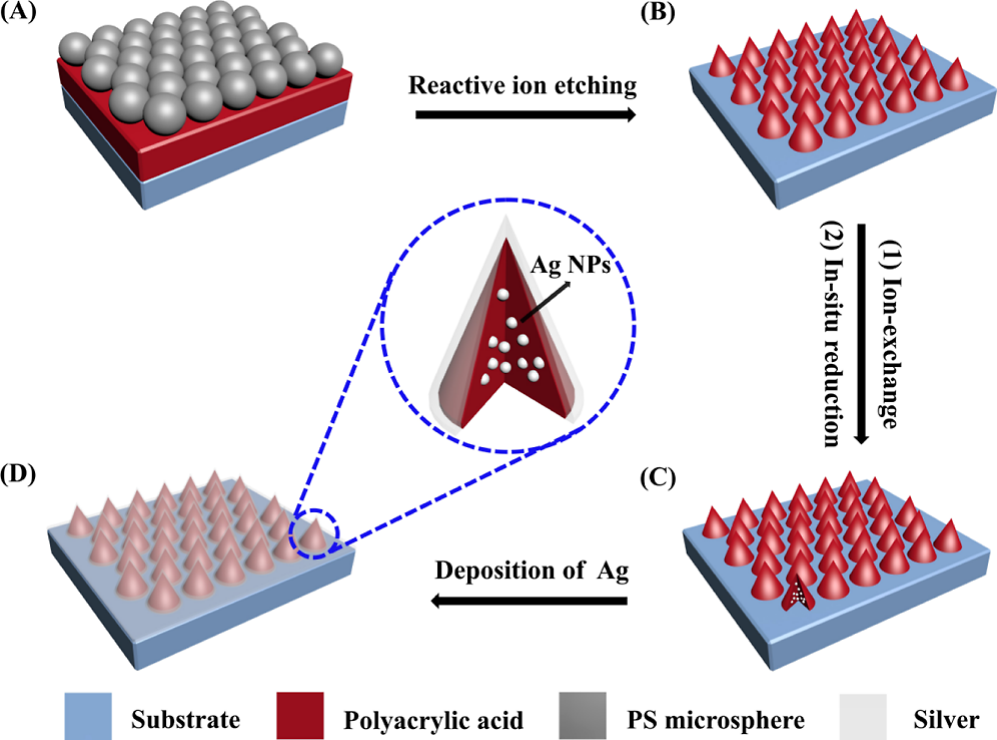
The manufacturing of an Ag nanocone array embedded with Ag NPs is depicted schematically. After spin-coating a PAA film onto a silicon substrate, hexagonal close-packed PS nanospheres were transferred to the surfaces of the PAA using the interfacial technique (A). The nanospheres were then totally etched using RIE, resulting in a PAA sheet with a periodic nanocone array (B). Afterward, Ag NPs were integrated into the PAA nanocones through ion exchange and in situ Ag^+^ reduction (C). Subsequently, a vertical silver film was put onto the substrate (D).

First, spin-coating was used to create the polyacrylic acid (PAA) film on a silicon substrate. By lifting PS nanospheres, such as those with a diameter of 500 nm, at the air/water interface, a hexagonal-close-packed (hcp) colloidal monolayer was produced on the substrate above, just as it had been constructed (Figure 1A). Using an ICP–reactive ion etching (RIE) DSE200S system, the colloidal sphere monolayer was etched in a moderate O_2_ plasma to remove the PS nanospheres and create the nanocone array of the PAA while preserving the original lattice positions (Figure 1B). Three crucial factors that affect the morphology of nanocones are the gas ratio, the pressure, and the power. Therefore, the etching process was carried out at 20 mTorr of pressure, 100 sccm of flow rate, 300 W of Src RF power, and 100 W of Bis RF power.

### 2.3 Fabrication of the Ag Nanocone Array Embedded with Ag NPs

The PAA nanocone array was immersed in a 0.3 M aqueous solution of AgNO_3_ for 12 h and then rinsed with Milli-Q water to prepare the PAA nanocone array embedded with Ag NPs. By being exposed to ultraviolet (UV) light, the Ag^+^ ions exchanged into the PAA nanocones were converted into Ag NPs (Figure 1C). Subsequently, an Ag film was successively deposited onto the nanocone array (Figure 1D). A thickness of 100 nm of Ag was chosen for the step’s deposit to allow adequate surface reflectance. Using this method, it is simple to create a large-area, highly ordered Ag nanocone array that is embedded with Ag NPs.

### 2.4 Simulations Using the FDTD

The electromagnetic field simulations were carried out using a commercial software program (FDTD Solutions v8.6.3, Lumerical Solutions Inc.) using identical structural features derived from the experimental samples. A plane wave of unit magnitude that was incident ordinarily moving in the *z*-direction while polarized along the *x*-axis activated such structures. The manual by Johnson and Christy was used to find the optical characteristics of Ag.

### 2.5 Analysis of the Solvent

The Ag nanocone array embedded with Ag NPs was used in the trial and its initial spectrum was recorded in the air. Next, a liquid cell was filled with a solvent and its resulting spectrum was recorded. Finally, the cell was purged with nitrogen to recover its initial range before the procedure was repeated with another solvent. To alter the RI of the surrounding medium, samples were submerged in methanol, ethanol, isopropanol, and dichloromethane. Every solvent shift was entirely reversible.

### 2.6 Characterization

Using a field-emission scanning electron microscope (Sigma, Carl Zeiss, Germany), the nanocone’s morphology and structure were examined. Thin coatings of gold were applied to all samples for scanning electron microscopy (SEM) analysis. A collimated beam of a fiber-coupled tungsten-bromine lamp (Ocean Optics) was used to measure the reflection spectra. The spectra were collected using an Ocean Optics USB4000 spectrometer with a wavelength range of 200–1100 nm. Transmission electron microscopy (TEM) was used to examine the morphology of the Ag NPs (TEM, model XL 30 ESEM FEG scanning electron microscope, FEI Company). The SERS spectra were measured using a Raman microscope (LabRAM HR Evolution) with a 633 nm excitation source (5% transmittance) and a 1 μm beam spot.

## 3 Results

### 3.1 Preparation of the PAA Nanocone Array

Figure 1 illustrates the overall fabrication process schematically. The polymer nanocone array was created using highly refined colloidal lithography.^47−49^ In summary, using an interfacial technique, hexagonal close-packed PS nanospheres with a diameter of 500 nm were applied to the surface of a silicon substrate with a layer of PAA (Figure 1A). The shape of a large-area hcp PS colloidal crystal is depicted in Figure S1 in the Supporting Information material (ESM). The PS nanosphere has a diameter of around 500 nm. Next, a RIE procedure was carried out (Figure 1B). The depth of the etched PAA film increases steadily as the etching duration increases.

**Figure 2:**
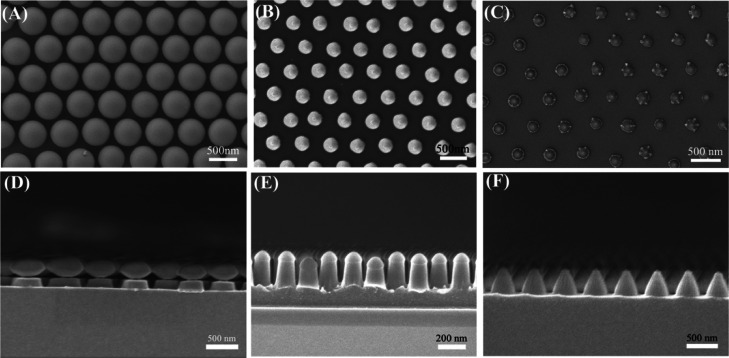
Morphological characterization for the etching process. The top-view SEM images and the SEM cross-section images of etching for 45 s (A,D), 90 s (B,E), and 135 s (C,F).

In contrast, the exposed PAA film is selectively removed by RIE, and the diameter of the nanosphere gradually decreases. Following the complete removal of the PS nanosphere, the PAA underwent synchronic etching, which produced periodic nanocone formations. Figure 2 demonstrates the corresponding morphological characterization for the etching process. Scanning electron microscopy (SEM) photographs of the etching process are presented in Figure 2A−C in the top view and Figures 2 D−F in the cross-section. These results indicate the successful fabrication of the PAA nanocone array.

### 3.2 Preparation of the Ag Nanocone Array Embedded with Ag NPs

**Figure 3:**
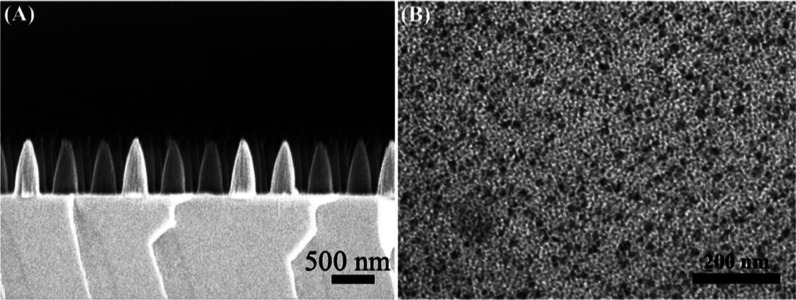
(A) SEM image of the PAA nanocone array embedded with Ag NPs. (B) TEM image of Ag NPs.

The fabrication of Ag nanoparticles was carried out using PAA nanocones as the matrix. Ag^+^ entered the swollen PAA network due to the ion exchange with the H^+^ in the swollen polymer network when the PAA equilibrated in an aqueous solution of AgNO_3_. Subsequently, UV irradiation resulted in the formation of Ag NPs (Figure 3A)^37,38^ (Figure 1C). Because it is difficult to identify the Ag NPs in Figure 3A, we made Ag NP/PAA composite films using the procedure described above to validate the existence of the Ag NPs inside the nanocones. The Ag NPs were generated with a mean diameter of about ∼5 nm, as demonstrated by a TEM picture (Figure 3B) and a narrow size distribution (Figure S2), which reveals that the Ag NPs were present inside the nanocones.

Entire and well-preserved silver nanocone structures were produced by sputtering a layer of silver film with a thickness of 100 nm onto the previously manufactured PAA/Ag NPs nanocone array (Figure 1D). Eventually, an array of Ag nanocones with Ag NPs was produced.

**Figure 4:**
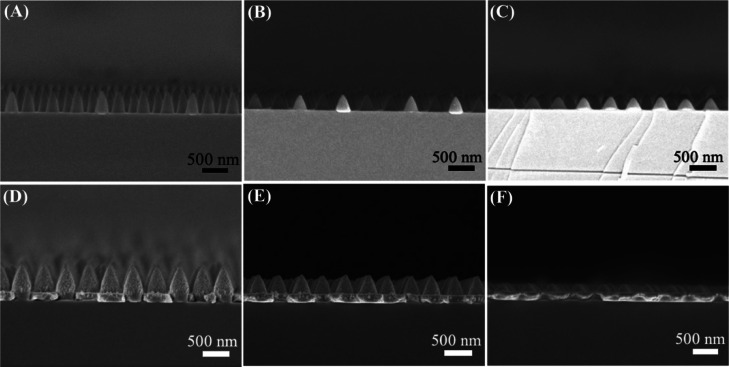
SEM images of nanocone arrays (A–C) before and (D–F) after the deposition of silver. The aspect ratios of nanocones are 1.47 (A,D), 1 (B,E), and 0.69 (C,F).

While keeping the lattice constant fixed at 500 nm, we built nanocone arrays with a range of heights and base diameters by adjusting the etching time of colloidal masks. Figure 4 illustrates a sequence of cross-sectional views of nanocone arrays before and after silver deposition, revealing a large-scale, very successful production. Figure 4A−C show the SEM images of nanocone arrays with average heights of approximately 560, 350, and 230 nm and average base diameters of about 380, 350, and 330 nm, respectively. These as-formed nanocones’ aspect ratios are 1.47, 1, and 0.69, respectively. Then, in Figure 4D–F, the Ag nanocone arrays containing Ag NPs were displayed. The producing silver film typically has a thickness of about 100 nm. The technique, as mentioned above, demonstrated good applicability for constructing nanocone array structures with different parameters.

### 3.3 Optical Properties of the Ag Nanocone Array Embedded with Ag NPs

Following the structural characterization studies, optical measurements were performed. The reflectance spectra were obtained from the nanocone array embedded with Ag NPs, Ag nanocone array, and the Ag nanocone array embedded with Ag NPs using a normal-incidence configuration. The observed reflectance spectra for the PAA nanocone array with Ag NPs implanted in it and the Ag nanocone array with the same average thickness (100 nm) are shown in Figure S3. The reflection spectrum of the nanocone array embedded with Ag NPs possesses distinct feature troughs at ∼420 nm, formed from the LSPR of Ag NPs.^50^ The SPP of the Ag nanocone array is responsible for the distinct highlighted troughs in the reflection spectrum of the Ag nanocone array at ∼500 nm.

**Figure 5:**
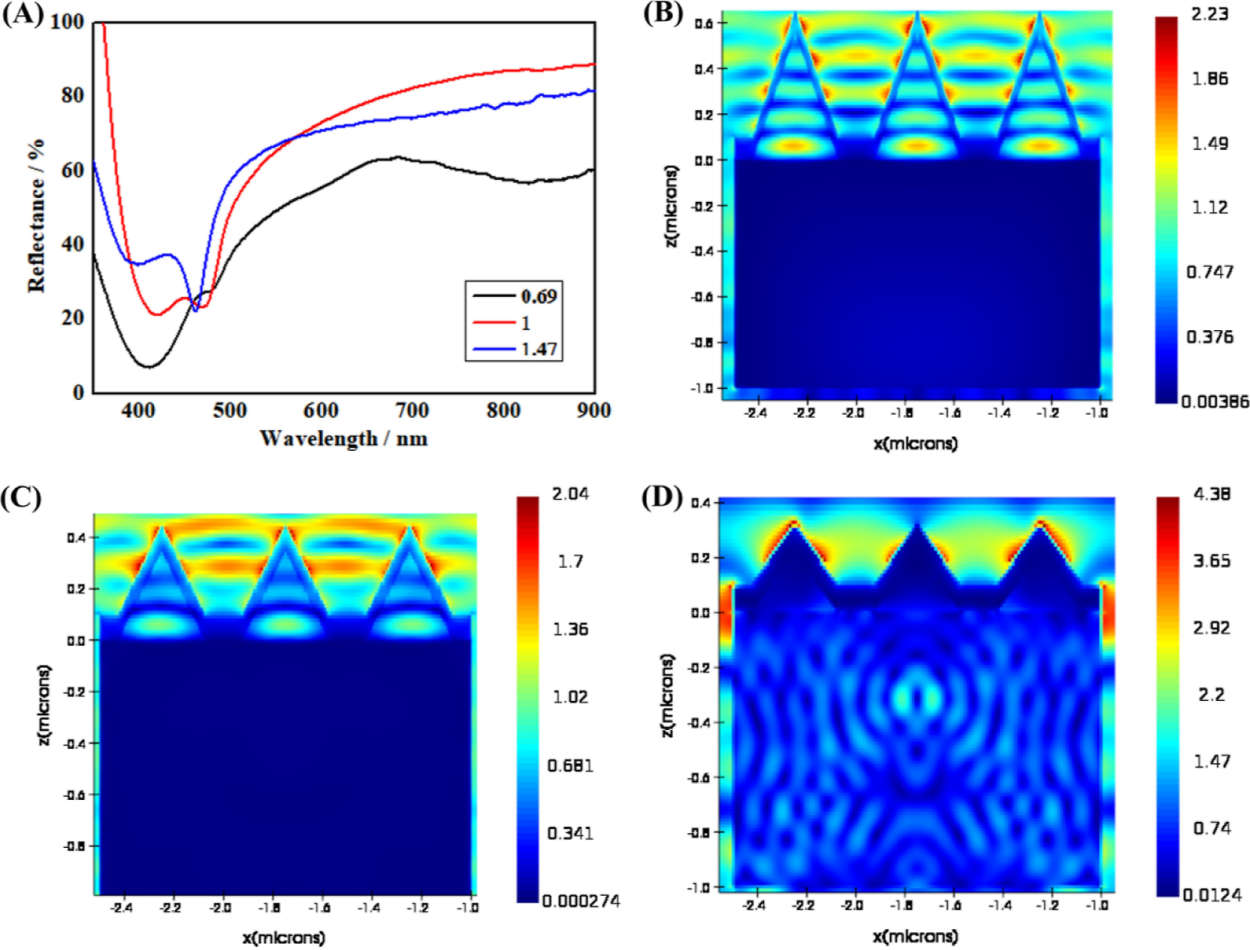
(A) Reflection spectra of the nanocone array embedded with Ag NPs for three different aspect ratios of nanocones: 0.69, 1, and 1.47. The field distribution diagram of Ag nanocone arrays with aspect ratios of 1.47 (B), 1 (C), and 0.69 (D) at the respective trough wavelength using FDTD simulation. A regularly incident, unit magnitude plane wave that was polarized along the *x*-axis and was moving in the *z*-direction simulated the structures.

The reflection spectra of the Ag nanocone array embedded with Ag NPs show several distinct features based on different aspect ratios (Figure 5A). However, they all possess two separate featured troughs, and the two troughs are between 420∼500 nm. Therefore, we believe that the troughs at ∼420 nm are attributed to Ag NPs inside the nanocone, and the characteristic troughs at ∼500 nm are attributed to the Ag nanocone arrays. The reflection spectra of the Ag nanocone array embedded with Ag NPs originate from the high coupling efficiency of the LSPR of Ag NPs inside the nanocone and the SPP of the Ag nanocone array. Importantly, the spectra indicate how the aspect ratio affects the featured troughs. When the aspect ratio of the nanocone is 1.47, the troughs at ∼500 nm are more pronounced than the troughs at ∼420 nm. The Ag nanocone array has a greater effect on the spectrum than the Ag NPs inside the nanocone. However, when the aspect ratio of the nanocone is 0.69, the troughs at ∼420 nm are more pronounced than the troughs at ∼500 nm. The Ag NPs inside the nanocone have a greater effect on the spectrum than the Ag nanocone array. However, there is not much difference between the troughs at ∼500 nm and the troughs at ∼420 nm when the aspect ratio of the nanocone is 1. In other words, the effect of the Ag nanocone array on the spectrum increases gradually with the aspect ratio of the nanocone. At wavelengths around 500 nm, two unique spectral evolution characteristics can also be seen. The wavelength of blue changes as the size of the nanocone rises and the troughs sharpen.

FDTD simulations of Ag nanocone arrays with various aspect ratios for the electric field distribution have been carried out to better illustrate the general evolution trend. As demonstrated in Figure 5B–D, the findings indicate that the Ag nanocone’s tip area is dominated by electric fields, with Figure 5B,C displaying significant coupling efficiency but rarely Figure 5D. This simulated result further explained the difference in the above spectra of nanocones with different aspect ratios.

### 3.4 Sensor Application of the Ag Nanocone Array Embedded with Ag NPs

**Figure 6:**
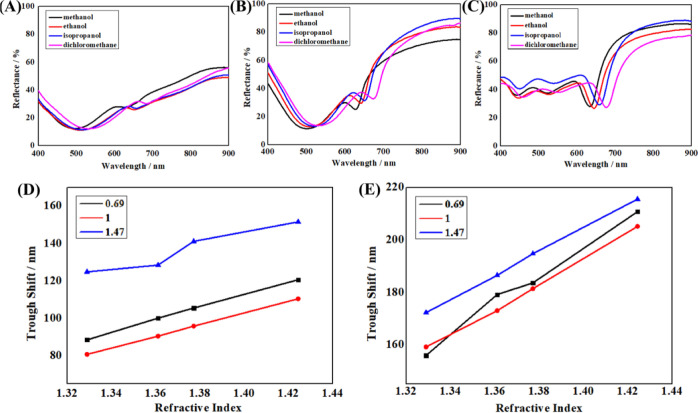
Reflectance spectra of the Ag nanocone array embedded with Ag NPs when immersed in solvents with different RIs. The aspect ratios of nanocones are 0.69 (A), 1(B), and 1.47 (C). (D) Relationship between the left trough shift (LSPR of Ag NPs) and the RI of the solvent. (E) Relationship between the right trough shift (SPP of the Ag nanocone array) and the RI of the solvent. The trough shift is the trough position difference obtained from air and solvent samples.

After that, samples immersed in different solvents had their reflectance spectra measured to study the refractive index sensitivity (RIS). Figure 6A–C displays the reflectance spectra of an Ag nanocone array implanted with Ag NPs and submerged in solvents of varying RI. The results reveal that the left trough (LSPR of Ag NPs) and right trough (SPP of the Ag nanocone array) all shift while the sharpness remains constant. Following that, Figure 6D,E plots the wavelength shifts for an Ag nanocone array (three different aspect ratios) embedded with Ag NPs as a function of the solvent’s refractive index. The wavelength shift shows where the trough wavelength is concerning the air in a solvent. The samples with aspect ratios of 0.69 and 1 display exact linear connections between trough shift and refractive index for the left trough (Figure 6D).

Other models with varying aspect ratios show varied relationships. While the samples with aspect ratios of 1.47 exhibit no linear relationships between trough shift and refractive index. The samples with aspect ratios of 1 and 1.47 show flawless linear correlations between trough shift and refractive index for the right trough. While the models with aspect ratios of 0.69 exhibit no linear relationships between trough shift and refractive index (Figure 6E). The above experimental data agree with the simulated results (Figure 5). The left troughs result from the LSPR of Ag NPs, and the right channels result from the SPP of the Ag nanocone array. Also, the RIS is closely related to the aspect ratio of the nanocone. When a nanocone array with an aspect ratio ≤1, Ag NPs inside the nanocone play a major role in the reflectance spectra. When a nanocone array with an aspect ratio ≥1, Ag nanocone arrays play a major role in the reflectance spectra. This study obtains the perfect RIS for the nanocone array with an aspect ratio of 1. The left trough shift (LSPR of Ag NPs) and the right trough shift (SPP of Ag nanocone array) both exhibit perfect linear relationships with refractive index. The current high-performance sensing platform may be possible with this appealing feature.

To verify the reliability of the research results. To study the signal improvements experimentally, we acquired SERS spectra on the Ag nanocone array embedded with Ag NPs for three distinct aspect ratios of nanocones (Figure S4). PATP, a nonresonant chemical, was chosen as the analyte because it can self-assemble into a monolayer on surfaces of silver film. The self-assembled monolayer of PATP molecules, which were coated on the Ag nanocone array embedded with Ag NPs for three distinct aspect ratios of nanocones via chemisorption, is shown in Figure S4 as SERS spectra in the region of 800–1800 cm^–1^. The incident laser was unpolarized and had a wavelength of 633 nm. Numerous low-intensity bands can be found in the spectra of PATP, in addition to the two prominent bands centred at 1077 and 1590 cm^–1^ that is easily distinguishable. From these observations, we conclude that the maximum at the aspect ratio is 1 due to the high couple effect of the Ag nanocone array and the LSPR of Ag NPs inside the nanocones, in accordance with the simulations mentioned above and experiments.

## 4 Conclusions

In conclusion, we established a unique colloidal lithography-based approach for producing Ag nanocone arrays embedded with Ag NPs as a high-performance plasmonic sensor. Using flexible colloidal lithography, the nanocone height and base diameter were logically and easily tuned based on the relationship of Ag nanocone array-SPP and Ag NPs-LSPR. The aspect ratio of nanocones was analyzed to manage the “SPP–LSPR” interaction. This impact significantly contributed to the sharpness and intensity of the line shape. We simulated the electric field profiles of Ag nanocone arrays with different aspect ratios by FDTD. The electric field has a strong intensity in the nanocones’ tip region. The larger aspect ratio exhibits the coupling effect of adjacent nanocones, while the smaller aspect ratio shows a much weaker coupling effect. Furthermore, we performed well-studied solvents with various refractive indices on the samples with different aspect ratios. The outstanding results indicate that the nanocone array with an aspect ratio of 1 is a potential detection platform.

## Supporting Information Available

Additional SEM images of the large-area hcp PS colloidal crystal; Ag NP size distribution; reflection spectra of the PAA nanocone array embedded with Ag NPs and the Ag nanocone array; and SERS spectra (PDF)
