# Supplementary material for: Rational Fabrication of Ag Nanocone Arrays Embedded with Ag NPs and Their Sensing Applications
Source: ACS Omega. 2022 Dec 6;7(50):46769–76. doi: 10.1021/acsomega.2c05854 (PMC9773957; doi:10.1021/acsomega.2c05854)
Supplement: Supplementary file 1 — ao2c05854_si_001.pdf [file ao2c05854_si_001.pdf]

Rational Fabrication of Ag Nanocone Array Embedded with Ag NPs and Sensing Applications

Hongxu Chen[1,2,3]*, Xing Li*[3]*, Yu Wang*[2]*, Yan Li*[2]*, Yingfeng Yu*[2]*, Haidong Li**[*,2]*, Baoqing Shentu**[*,1]

[1]State Key Lab of Chemical Engineering, Department of Chemical and biological Engineering, Zhejiang University, Hangzhou 310027, China

[2]College of Material and Textile Engineering, Jiaxing University, Jiaxing 314001, China

[3]Zhejiang Yuhua Timber Co., LTD, Jiaxing 314101, China

SUPPLEMENTARY FIGURES

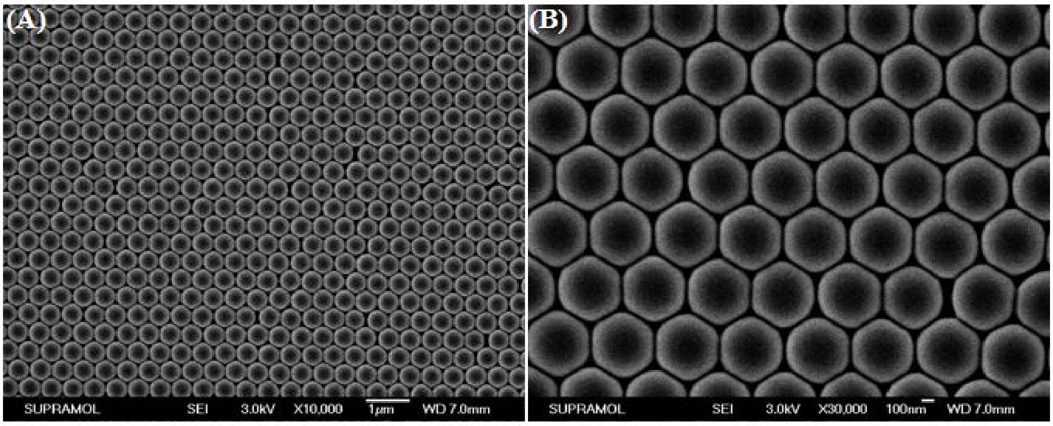

Figure S1. SEM images of large-area hcp PS colloidal crystal at low magnification (A) and at high magnification (B).

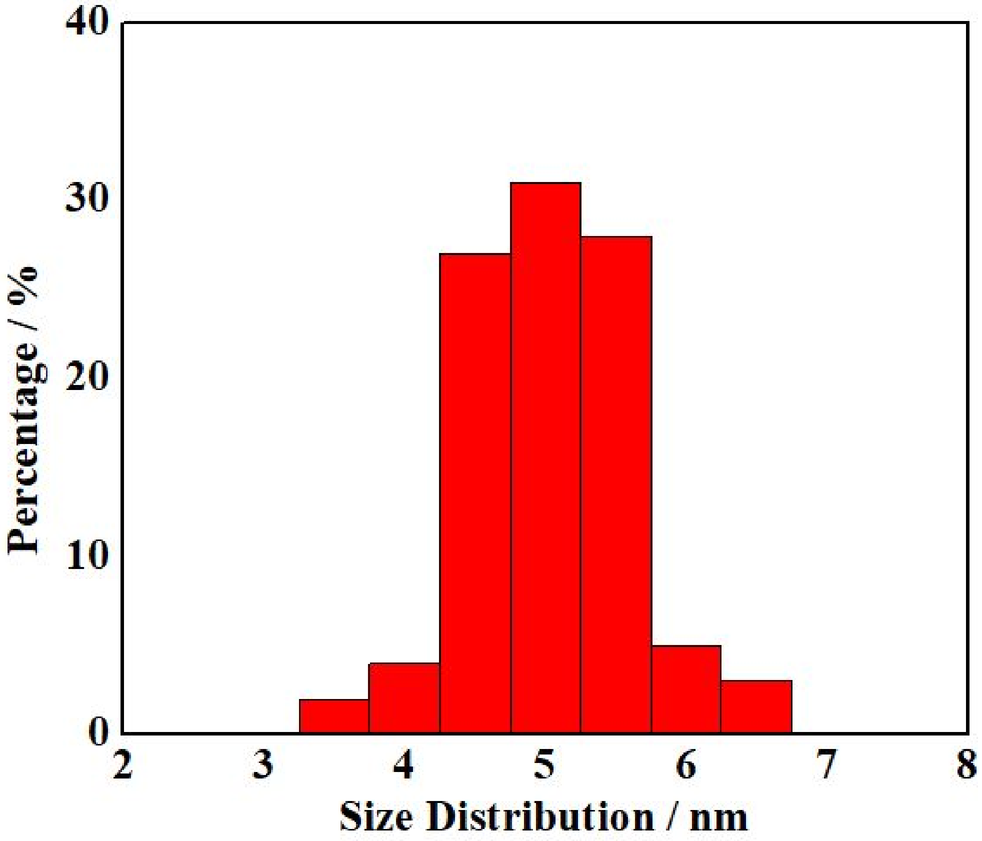

Figure S2. Ag NP size distribution based on parameters from 100 Ag NPs.

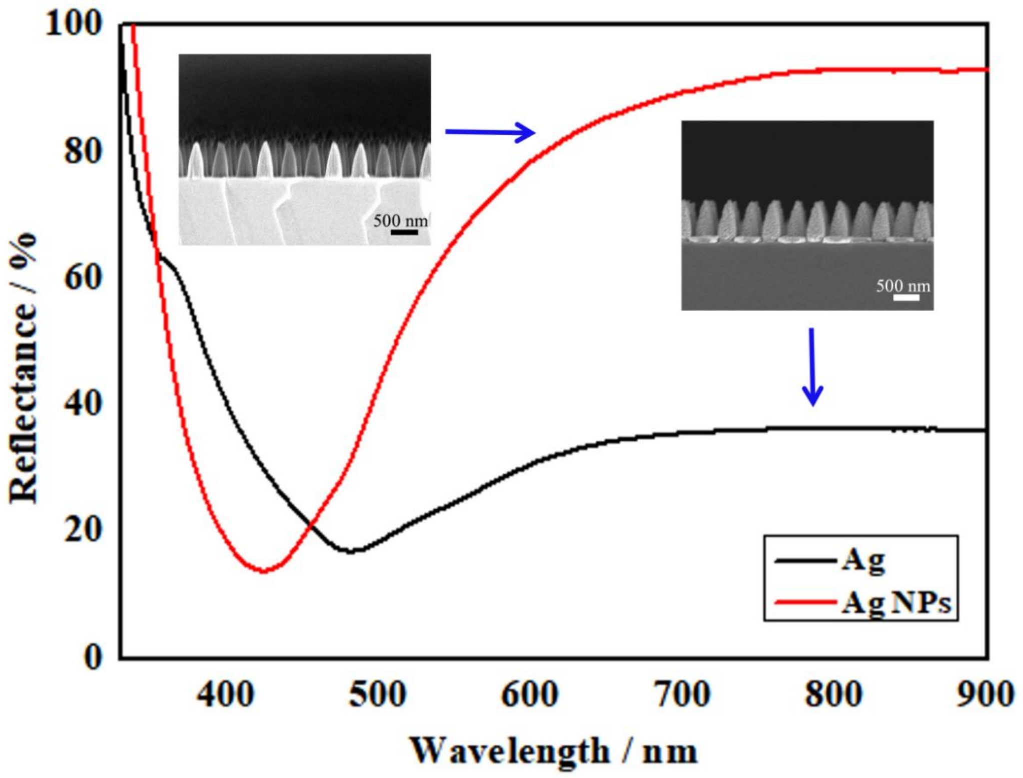

Figure S3. The reflection spectra of the PAA nanocone array embedded with Ag NPs (red line) and Ag nanocone array without Ag NPs (black line).

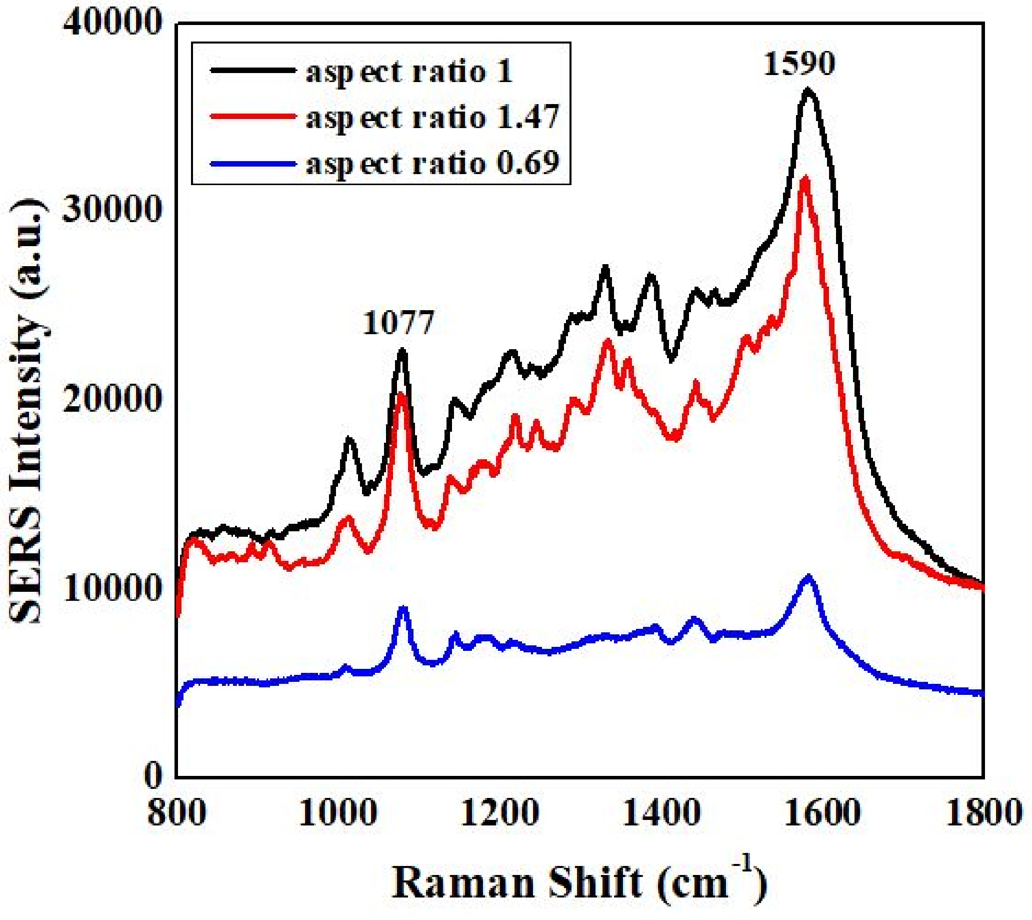

Figure S4. SERS spectra in the range of 800–1800 cm^{-1} for a self-assembled monolayer of PATP molecules, which were deposited on the Ag nanocone array embedded with Ag NPs for three different aspect ratios of nanocones.
